# Drought legacies and ecosystem responses to subsequent drought

**DOI:** 10.1111/gcb.16270

**Published:** 2022-06-23

**Authors:** Lena M. Müller, Michael Bahn

**Affiliations:** ^1^ Department of Ecology University of Innsbruck Innsbruck Austria

**Keywords:** drought legacy, drought recovery, drought response, lagged effects, legacy duration, post‐drought state, recurrent drought, resilience

## Abstract

Climate change is expected to increase the frequency and severity of droughts. These events, which can cause significant perturbations of terrestrial ecosystems and potentially long‐term impacts on ecosystem structure and functioning after the drought has subsided are often called ‘drought legacies’. While the immediate effects of drought on ecosystems have been comparatively well characterized, our broader understanding of drought legacies is just emerging. Drought legacies can relate to all aspects of ecosystem structure and functioning, involving changes at the species and the community scale as well as alterations of soil properties. This has consequences for ecosystem responses to subsequent drought. Here, we synthesize current knowledge on drought legacies and the underlying mechanisms. We highlight the relevance of legacy duration to different ecosystem processes using examples of carbon cycling and community composition. We present hypotheses characterizing how intrinsic (i.e. biotic and abiotic properties and processes) and extrinsic (i.e. drought timing, severity, and frequency) factors could alter resilience trajectories under scenarios of recurrent drought events. We propose ways for improving our understanding of drought legacies and their implications for subsequent drought events, needed to assess the longer‐term consequences of droughts on ecosystem structure and functioning.

## Graphical abstract

Climate change increases the frequency and severity of drought events, which can have lasting impacts on ecosystem structure and functioning after the drought has subsided. In this review, we synthesize the current knowledge on drought legacies and the underlying mechanisms, highlight the relevance of legacy duration for different ecosystem processes on the examples of carbon cycling and community composition, and develop hypotheses on how intrinsic (i.e. biotic and abiotic properties and processes) and extrinsic (i.e. drought timing, severity, and frequency) factors could alter resilience trajectories under scenarios of recurrent drought events.

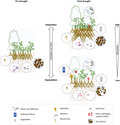

## 1 INTRODUCTION

Climate change has been and will likely be causing a significant increase in the severity and frequency of drought events (IPCC, 2021; Spinoni et al., 2018; Trenberth et al., 2014) with strong repercussions on ecosystem processes and services (Bastos et al., 2020; Ciais et al., 2005; Feeley et al., 2020; Reichstein et al., 2013; Thonicke et al., 2020; Vicente‐Serrano et al., 2020). In addition to the concurrent effects of drought events on ecosystems, manifold changes can persist after the drought has subsided (Frank et al., 2015). These post‐drought effects are commonly referred to as “drought legacies” (Vilonen et al., 2022) and have been demonstrated for various aspects of ecosystem structure and functioning. Drought legacy effects have been associated with altered carbon (C) cycling (Craine et al., 2013; Kannenberg et al., 2020; Liu et al., 2022; Scott et al., 2010; Wei et al., 2022; Xie et al., 2020), nitrogen (N) cycling (DeLong et al., 2019; DeVries et al., 2012; Legay et al., 2018), growth (Anderegg, Schwalm, et al., 2015; Gazol et al., 2020; Wu et al., 2018; Zhao et al., 2020), phenology (Berwaers et al., 2019; Hoover et al., 2021; Kang et al., 2018; Peng et al., 2019; Sippel et al., 2018; Zeng et al., 2021), species composition (DeBoeck et al., 2018; Griffin‐Nolan et al., 2019; Stampfli et al., 2018; Stampfli & Zeiter, 2020; Winkler et al., 2019), herbivory (Gutbrodt et al., 2011), as well as soil physicochemical properties (Goebel et al., 2011; Sánchez‐García et al., 2019). Drought legacies have also been associated with increased plant mortality (Bigler et al., 2007; Hammond, 2020; Hartmann et al., 2018; Sippel et al., 2018; Trugman et al., 2018; Zhou et al., 2019), and with reduced plant defense against pests and pathogens (Jactel et al., 2012; Trugman et al., 2021; Wiley et al., 2016).

All these biotic and abiotic legacies from species to ecosystem scale are summarized below and referred to as legacies in intrinsic factors. In addition to these intrinsic factors a range of extrinsic factors, including drought *timing*, drought *severity* (intensity and duration), and drought *frequency* can affect drought legacies.

Although the relevance of drought legacies for a longer‐term perspective on ecosystem resilience (the resistance to and recovery from subsequent drought events (Ingrisch & Bahn, 2018; Lloret et al., 2011) has been increasingly acknowledged in recent years (Anderegg et al., 2020; Canarini et al., 2021; DeSoto et al., 2020; Hahn et al., 2021), our understanding of drought legacies and the underlying processes is still restricted to a few case studies or specific aspects of plant and ecosystem functioning, such as radial tree growth (see Figure S1; Gazol et al., 2020; Kannenberg et al., 2020; Kannenberg, Novick, et al., 2019). Hence, we still lack a clear understanding of how drought legacies alter the resilience of ecosystems to subsequent drought events. This is of particular relevance given that drought frequency is likely to increase in the coming decades (IPCC, 2021; Wang, Tu, et al., 2021).

This review aims to (i) synthesize our current understanding of drought legacies and the underlying mechanisms from species and communities to ecosystem (biotic and abiotic) scale and (ii) summarize the legacy duration of previously documented drought legacy responses. Furthermore, we (iii) develop hypotheses as to how drought legacies of intrinsic factors could influence the resilience trajectories of ecosystem responses to subsequent drought events relating to extrinsic factors.

## 2 DEFINING AND CHARACTERIZING DROUGHT LEGACIES

Drought legacies are commonly defined as any alterations of an ecosystem state or processes that occur after a drought has subsided (Buttlar et al., 2018; DeBoeck et al., 2018; Delgado‐Balbuena et al., 2019; Griffin‐Nolan et al., 2018; Rousk et al., 2013; Sala et al., 2012; Vilonen et al., 2022; Walter et al., 2013). They refer to changes of intrinsic factors after a disturbance event (see material legacies [Johnstone et al., 2016]) compared to evolutionary adaptions to historical disturbance regimes (see information legacies [Johnstone et al., 2016]). Drought legacies can involve both reductions and enhancements in response parameters (Frank et al., 2015; Griffin‐Nolan et al., 2018; Sala et al., 2012).

Next to the term ‘drought legacy’ several other terms have been used in the literature, including ‘lagged effects’ (Zhao et al., 2018), ‘stress imprint’ (Bruce et al., 2007), ‘stress memory’ (Fleta‐Soriano & Munné‐Bosch, 2016; Walter et al., 2013) or ‘drought memory’ (Canarini et al., 2021; Ogle et al., 2015; Walter et al., 2011; for a broader discussion see also [Vilonen et al., 2022]).

**FIGURE 1:**
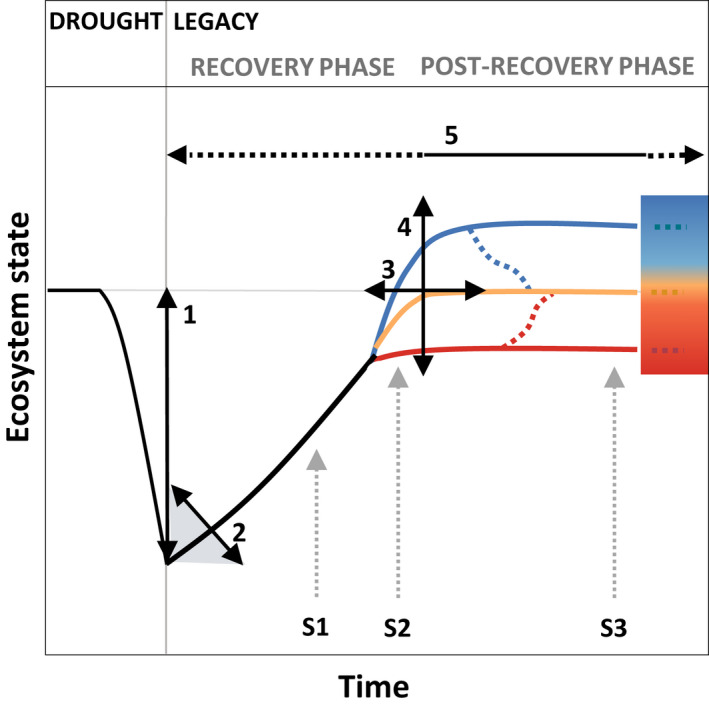
Post‐drought trajectories of the recovery and post‐recovery phase after a drought event. The recovery phase is characterized by the rate of recovery (arrow 2) following the maximum impact of the drought event (arrow 1). The post‐recovery phase starts when the rate of recovery is zero (arrow 3), irrespective of whether the recovery has been complete (yellow trajectory) or has resulted in a shifted baseline, the latter reflecting an immediate drought legacy (red and blue trajectories). In the post‐recovery phase drought legacies can be characterized by the deviation from the pre‐drought baseline (arrow 4) and the legacy duration (arrow 5). Starting timepoints (S1–S3) of a potential subsequent drought event (see Figure 4) are indicated as dotted gray arrows.

**FIGURE 2:**
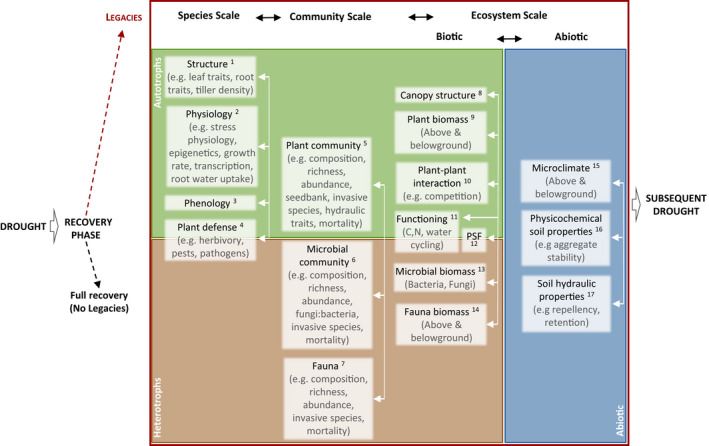
Drought legacies on species, community, and ecosystem scale. Colors refer to autotrophic (green), heterotrophic (brown), and abiotic (blue) ecosystem components, respectively. White arrows indicate interactions of legacies across properties within a given scale (cross‐scale interactions not shown for simplicity). See Figure S1 for the number of papers published on the respective topics. Respective key references are indicated as follows, and for additional references, see manuscript: 1. Reichmann et al. (2013), DeVries et al. (2016), Delgado‐Balbuena et al. (2019), Griffin‐Nolan et al. (2019), Metz et al. (2020); 2. Bruce et al. (2007), Ding et al. (2012), Kinoshita and Seki (2014), Crisp et al. (2016), Darenova et al. (2017), Fleta‐Soriano and Munné‐Bosch (2016), Kannenberg, Novick, et al. (2019), Kannenberg et al. (2020), Zhao et al. (2020); 3. Kang et al. (2018), Berwaers et al. (2019), Peng et al. (2019), Hoover et al. (2021), Zeng et al. (2021); 4. Jactel et al. (2012), Anderegg, Hicke, et al. (2015), Kolb et al. (2016), Schlesinger et al. (2016); 5. Anderegg et al. (2013), Hoover et al. (2014), Frank et al. (2015), Clark et al. (2016), Xu et al. (2017), DeBoeck et al. (2018), Sippel et al. (2018), Stampfli et al. (2018), Griffin‐Nolan et al. (2019), Winkler et al. (2019), Batllori et al. (2020), Brodribb et al. (2020), Wilcox et al. (2021); 6. Kaisermann et al. (2017), DeVries et al. (2018), Meisner et al. (2018), Preece et al. (2019), Valliere et al. (2019), Kelso et al. (2020), Wang and Allison (2021), Liu et al. (2022); 7. Lindberg and Bengtsson (2006), Coyle et al. (2017); 8. Saatchi et al. (2013), Kannenberg, Novick, et al. (2019), Jiao et al. (2021), Senf et al. (2021); 9. Griffin‐Nolan et al. (2018), Sala et al. (2012), Petrie et al. (2018), DeVries et al. (2016), Yang et al. (2018), DeVries et al. (2019), Wigneron et al. (2020); 10. Stampfli et al. (2018), Kaisermann et al. (2017); 11. DeVries et al. (2012), Acosta‐Martinez et al. (2014), DeVries et al. (2016), DeVries et al. (2018), Legay et al. (2018), Meisner et al. (2018), DeLong et al. (2019), Huang et al. (2017), Berwaers et al. (2019), Delgado‐Balbuena et al. (2019), Mackie et al. (2019), Ji et al. (2021), Dong et al. (2021), Hoover et al. (2021), Liu et al. (2022); 12. van der Putten et al. (2013), Preece and Peñuelas (2016), van der Putten et al. (2016), Kaisermann et al. (2017), Sasse et al. (2018), DeVries et al. (2019), Peguero et al. (2019), Pugnaire et al. (2019), Crawford and Hawkes (2020), Sánchez‐Cañizares et al. (2017); 13. DeLong et al. (2019), Dong et al. (2021), Liu et al. (2022); 14. DeVries et al. (2012), Coyle et al. (2017); 15. Kane et al. (2011), Royer et al. (2011), Anderegg et al. (2012), Anderegg et al. (2013); 16. Goebel et al. (2005), Goebel et al. (2011); 17. Robinson et al. (2016), Sánchez‐García et al. (2019).

In this paper, we use the term drought legacy to describe any shift in ecosystem properties or processes after a drought has subsided (Figure 1). Thus, drought legacies include both the recovery phase after the drought has ended and the post‐recovery phase, in the case of incomplete recovery (Figure 1). The recovery phase is characterized by the rate of recovery (arrow 2) following the maximum impact of the drought event (arrow 1). The post‐recovery phase starts when the rate of recovery levels off (arrow 3), and the recovery is complete (yellow trajectory, no legacy) or incomplete, that is the baseline has been shifted (red and blue trajectories, legacy). These shifts can occur on all organizational scales, including species, community and/or ecosystem (Figure 2).

Given that the ecosystem state changes dynamically during the recovery phase, the most coherent approach to quantifying and comparing drought legacies would be to compare the ecosystem post‐recovery state with the baseline state (see below; Figure 1). However, this may be difficult to achieve in cases when recovery rates are difficult to quantify, for example due to insufficient time resolution for assessing recovery dynamics, or due to intrinsic factors, which recover very slowly, for example community properties, which can take years or decades to recover fully (Albertson & Weaver, 1944; Stampfli & Zeiter, 2004).

To date, different baselines of an ecosystem state, such as the pre‐disturbance level (Gazol et al., 2020), control (Arredondo et al., 2016; DeBoeck et al., 2018; Mackie et al., 2019; Yahdjian & Sala, 2006), or the predicted level (Anderegg, Schwalm, et al., 2015; Delgado‐Balbuena et al., 2019; Peltier et al., 2016; Wu et al., 2018) have been used to characterize drought legacies. We suggest a characterization of drought legacies post‐drought or, if possible, post‐recovery via the legacy duration and the legacy size (deviation from the pre‐drought, control, or the predicted baseline; Figure 1). While we acknowledge that pre‐ and post‐drought baselines can fluctuate strongly over time (Bahn & Ingrisch, 2018), we suggest that such a characterization can enhance comparability of drought legacies across ecosystems and response parameters in future studies, especially when combined with a consistent design of drought studies (Munson et al., 2020; Slette et al., 2019).

Given that drought legacies may have strong repercussions on ecosystem responses to subsequent droughts, we argue that a drought legacy should consider the whole timespan during which the ecosystem state and its responses to environmental conditions, including a subsequent drought, are altered by a drought event (see also Section 5).

## 3 DROUGHT LEGACIES AND THE UNDERLYING MECHANISMS AT SPECIES, COMMUNITY, AND ECOSYSTEM SCALES

Drought can have long lasting effects on intrinsic factors from species, community, to ecosystem scales (see Figure 2 and below). The legacy size and duration of these intrinsic factors can be affected by a range of extrinsic factors, including drought timing, drought severity (intensity and duration), and drought frequency. For example, drought *timing* can alter growth legacies in forests, such that the legacy size is higher in the later (Kannenberg, Maxwell, et al., 2019) or drier part of the growing season (Huang et al., 2018). In grasslands, the effects of drought *timing* on the size of growth legacies increase the later the drought occurs in the season (Hahn et al., 2021). Also drought *intensity* impacts the legacy size, which increases with increasing drought intensity (Kannenberg, Maxwell, et al., 2019; Yahdjian & Sala, 2006). Furthermore, a longer drought *duration* was observed to also enhance legacy duration (Jiao et al., 2021). Moreover, there is increasing evidence that ecosystem responses to drought *intensity* and *duration* are nonlinear during drought (Dannenberg et al., 2019; Felton et al., 2021; Wang, Vera‐Vélez, et al., 2021; Zhang et al., 2021), with potential consequences for drought legacies, though these remain to be explored.

In the following, we provide an overview of post‐drought legacies and the underlying mechanisms from species to community and ecosystem scales (broadly summarized in Figure 2).

### 3.1 Species scale

Drought can lead to a range of *structural* changes on the species scale. For example, in grasslands, drought can decrease the tiller and stolon density, with consequences for ecosystem productivity (Delgado‐Balbuena et al., 2019; Reichmann et al., 2013; Reichmann & Sala, 2014). Moreover, drought can reduce belowground bud density (Qian et al., 2022) as well as reproductive output with consequences for grassland community composition (Zeiter et al., 2016). Furthermore, drought can increase the number of seeds and decrease the number of leaves (Metz et al., 2020). Moreover, drought can induce a shift toward resource‐conservative root traits such as lower specific root length (DeVries et al., 2016) and can increase community‐weighted plant traits such as specific leaf area and leaf N content, which reflects a shift toward communities with drought avoidance and escape strategies (Griffin‐Nolan et al., 2019). After recovery from drought, increased shoot, root, and tissue N concentrations of herbaceous species have often been observed, which is probably due to higher post‐drought N availability (see also ecosystem section below; DeLong et al., 2019; Ingrisch et al., 2018; Roy et al., 2016). In forests, drought can lead to structural changes such as a decrease in active xylem area, as well as needle shedding or canopy loss (Peltier & Ogle, 2019).

On a *physiological* level, drought can alter the growth rate of species across plant functional types, and as a result, legacy effects can be positive or negative (Darenova et al., 2017; DeVries et al., 2012; Itter et al., 2019; Kannenberg et al., 2020; Kannenberg, Novick, et al., 2019; Li et al., 2020; Peltier & Ogle, 2019; Zhao et al., 2020). Drought legacies of tree ring studies predominantly suggest negative effects on growth (Anderegg, Schwalm, et al., 2015; Kannenberg et al., 2020). In trees, post‐drought reductions of root functioning (Peltier & Ogle, 2019) and an altered stomatal sensitivity to soil and plant water status (Grossiord et al., 2018) have been observed. Furthermore, drought can alter molecular mechanisms such as pathways of signalling metabolites, transcription factors, or epigenetics involving modifications in DNA, histone, or chromatin organization (Alves et al., 2020; Bruce et al., 2007; Crisp et al., 2016; Ding et al., 2012; Kinoshita & Seki, 2014; Sahu et al., 2013), with consequent structural changes, including short‐term changes such as the pigment composition of leaves (Fleta‐Soriano & Munné‐Bosch, 2016).

Drought legacies have also been associated with altered *phenology* both of herbaceous and woody species, for example earlier end‐of‐season senescence leading to a shortened growing season (Berwaers et al., 2019; Hoover et al., 2021; Kang et al., 2018; Peng et al., 2019). These effects are especially pronounced in regions with generally low water availability (Peng et al., 2019). Prior‐season drought (Zeng et al., 2021) and spring drought (Kang et al., 2018) can lead to a delay in the onset of spring growth and hence the start of the growing season, with negative impacts on summer growth rates (Zeng et al., 2021). Finally, drought can advance the flowering date and increase the flowering duration. The phenological response can vary depending on the species and the diversity of a stand with potential long‐term effects on reproductive fitness (Jentsch et al., 2009).

*Plant mortality* is a widespread drought legacy with significant consequences for the community and the ecosystem scale. Mortality can occur both during (Choat et al., 2018; Jung et al., 2020) and after a severe drought event (Anderegg et al., 2013; Anderegg, Hicke, et al., 2015; Bigler et al., 2007; Brodribb et al., 2020; Frank et al., 2015; Harrison et al., 2018; Schlesinger et al., 2016; Senf et al., 2020; Sippel et al., 2018; Stampfli et al., 2018; Trugman et al., 2018, 2020). Tree mortality has frequently been associated with hydraulic failure, but C limitation also has been discussed as a possible cause in some cases (Adams et al., 2017; Choat et al., 2018; Gessler et al., 2017; McDowell et al., 2020, 2022). Additionally, lags in soil water replenishment following drought (van der Molen et al., 2011) can enhance species mortality (Goulden & Bales, 2019). Furthermore, drought often leads to reduced *plant defense* against herbivory, pests, and pathogens, which increases the risk of plant mortality in trees and herbaceous species (Anderegg, Hicke, et al., 2015; Gaylord et al., 2013; Gutbrodt et al., 2011; Jactel et al., 2012; Kolb et al., 2016; Schlesinger et al., 2016; Trugman et al., 2021; Wiley et al., 2016).

### 3.2 Community scale

Drought can exert legacy effects on *plant communities* by reducing species richness (Stampfli et al., 2018), abundance of specific species (Hoover et al., 2014; Jung et al., 2014), and diversity (Xu et al., 2017), but drought has also been shown to increase functional diversity (Griffin‐Nolan et al., 2019). In grassland exposed to drought, plant composition shifted toward more stress‐resistant slower growing species (Wilcox et al., 2021). Results of single case studies performed in prairie (Hoover et al., 2014) or with alpine grassland mesocosms (DeBoeck et al., 2018) suggest that grasses are probably more drought resistant than forbs. In addition to different resistance to drought, community reorganization toward grass domination can also be driven by altered plant–plant interactions, such as competition, with resource‐acquisitive grasses dominating at the expense of resource‐conservative forbs (Stampfli et al., 2018). In contrast, droughts may favor an increase of forbs, which have been suggested to outperform grasses in their capacity to recruit from seed (Stampfli & Zeiter, 2004). In grasslands where shrubs are present, they can replace perennial grasses as a response to drought due to their more extensive root systems permitting access to deeper water (Winkler et al., 2019).

In forests, community reorganization following drought can lead to shifts in dominant tree species and their associated above‐ and belowground communities, involving shifts toward more drought tolerant and xeric communities and related traits, and in savannas shifts towards non‐woody vegetation (Anderegg et al., 2013; Batllori et al., 2020; Brodribb et al., 2020; Clark et al., 2016; Suarez & Kitzberger, 2008; Trugman et al., 2020). Community shifts can also be species‐unspecific, as for example mortality is often related to tree density and tree size, irrespective of the species involved (Brodribb et al., 2020; Cui et al., 2022; McDowell et al., 2020; Trugman et al., 2020).

Drought and rewetting have strong impacts on *soil communities*. Drought can alter species composition and generally tends to decrease the abundance and the richness of *soil fauna* (Coyle et al., 2017; DeVries et al., 2012; Lindberg et al., 2002; Lindberg & Bengtsson, 2006). It has recently been shown to also cause legacies in the *microbial community* composition (Canarini et al., 2021; Evans et al., 2022; Kaisermann et al., 2017; Liu et al., 2022; Meisner et al., 2018, 2021; Xi et al., 2022). Drought was observed to promote fungi and to reduce bacteria (Fuchslueger et al., 2014; Preece et al., 2019) and bacterial networks (DeVries et al., 2018). Drought can also alter microbial community‐level traits, but the magnitude and persistence of such drought legacies is under debate (Wang & Allison, 2021). Drought effects on plant–soil feedbacks, which can strongly alter above‐ and belowground communities, will be discussed in the ecosystem section.

Drought‐induced changes on the community scale can also be driven by *invasive species*. Generally, when invasive species are already established, they tend to negatively affect plant communities through a loss in plant diversity, shifted community composition, and a dampened recovery capacity of natives from drought (Fahey et al., 2018; Vetter et al., 2020; Xu et al., 2022). In invaded grassland plant communities, drought was observed to impact the growth of invasive species less (Meisner et al., 2013) or more (Valliere et al., 2019) compared to native species. When negatively affecting plant growth of invasives, drought can lead to a long lasting reduction in the presence of invasive plants post drought (Kelso et al., 2020). The effects of growth and reproduction can be weakened by higher germination rates of seeds of invasive compared to natives species (Valliere et al., 2019).

### 3.3 Ecosystem scale

Drought can lead to a range of legacies on the ecosystem scale, which can be driven by changes on species or community scale and can feed back to these scales.

Drought can induce pronounced legacy effects on ecosystem *C cycling*, for example through legacy effects on plant biomass (Wigneron et al., 2020; Yang et al., 2018) and biomass production. Drought legacy effects on aboveground net primary production (ANPP) can be positive (Griffin‐Nolan et al., 2018) or negative (Petrie et al., 2018; Sala et al., 2012). Enhanced post‐drought growth can compensate for the growth reductions during drought and stabilize overall biomass production (Hahn et al., 2021; Mackie et al., 2019; Stampfli et al., 2018). In grasslands, drought legacy effects on ANPP have been associated with tiller recruitment (Reichmann et al., 2013; Reichmann & Sala, 2014), changes in the composition of species and functional groups (DeBoeck et al., 2018; Gao et al., 2021; Hoover et al., 2014), as well as changes in nutrient availability (DeLong et al., 2019; Mackie et al., 2019). Drought can also lead to increased (Berwaers et al., 2019) or decreased carbon uptake and respiration (Delgado‐Balbuena et al., 2019), and affect soil respiration (Dong et al., 2021; Liu et al., 2022). Post‐drought changes in microbial biomass or in microbial community‐level traits can alter soil C cycling such as soil respiration (Dong et al., 2021; Evans et al., 2022; Liu et al., 2022) and soil organic matter decomposition (Wang & Allison, 2021). Furthermore, drought can have a positive or negative legacy effect on water use efficiency (WUE), that is the amount of C taken up relative to the amount of water lost (Huang et al., 2017; Ji et al., 2021; Yang et al., 2016). Generally, post‐drought changes in WUE last longer for forests (up to 1 year) than for shrubland and sparse vegetation (up to 4 months; Ji et al., 2021). In the longer term, changes in plant species composition after a drought event toward drought‐tolerant species has been suggested to increase C and water cycling (Craine et al., 2013).

Drought and rewetting can alter N cycling and the short‐term dynamics of *soil N availability*. Upon rewetting, large pulses in nutrient release and N mineralization can occur (Birch, 1958; Leitner et al., 2017; Manzoni et al., 2012; Schimel, 2018; van Sundert et al., 2020). This higher availability of N post‐drought was observed to enhance recovery of plant growth in grasslands (Ingrisch et al., 2018; Karlowsky et al., 2018; Roy et al., 2016; Schrama & Bardgett, 2016), thereby reducing potential subsequent plant growth legacies. Indeed, an increase in soil N following drought was found to be accompanied in grasslands by higher plant growth and in consequence biomass (DeLong et al., 2019; DeVries et al., 2012; Legay et al., 2018; Mackie et al., 2019). In forests, the higher nutrient supply post drought can enhance tree recovery, which strongly depends on the re‐establishment of root functions as well as root damage and mortality (Gessler et al., 2017). Furthermore, drought‐induced effects on roots as well as leaf senescence can affect nutrient status and nutrient demand post‐drought (Schlesinger et al., 2016). For example, N uptake under drought can be reduced (Joseph et al., 2021) and detrimental impacts of drought on K availability can reduce tree resistance to subsequent drought (Touche et al., 2022).

Post‐drought N availability can also be altered by changes in microbial communities (Meisner et al., 2018). For example, drought can select for microbial communities with a lower capacity to immobilize N which leads, together with lower root N uptake, to higher soil N concentration (DeVries et al., 2016). Also drought‐related changes in fungi/bacteria ratios can result in altered ecosystem N and C cycling (DeVries et al., 2018) and induce possible feedback to plants and alter plant–plant interactions (Kaisermann et al., 2017). Furthermore, drought legacy effects on N cycling in grasslands can be induced by a decrease in soil microbial activity post‐drought, as microbial enzymatic activities are highly sensitive to drought (Acosta‐Martinez et al., 2014; Legay et al., 2018).

A major driver of drought legacies in grasslands is related to drought‐induced changes in *plant–soil feedbacks* (PSFs), that is the interactions between plants, soil organisms, and abiotic soil factors, which lead to altered plant composition and performance and have cascading effects on ecosystem properties (Buchenau et al., 2022; Crawford & Hawkes, 2020; DeVries et al., 2019; Peguero et al., 2019; Preece & Peñuelas, 2016; Pugnaire et al., 2019; van der Putten et al., 2013; van der Putten et al., 2016; Williams & DeVries, 2020). Drought can influence PSFs e.g. via drought‐driven changes in the composition of plant species, whose roots interact with the respective symbionts, decomposers, and pathogens (Pugnaire et al., 2019; van der Putten et al., 2016). Similarly, drought can influence PSFs via changes in belowground community composition (Pugnaire et al., 2019; van der Putten et al., 2016). Thereby, drought‐induced changes in microbial communities can alter the direction and intensity of PSFs with consequences for ecosystem properties, for example by positively or negatively affecting plant growth (Kaisermann et al., 2017). Drought effects on PSFs can be mediated both in terms of quantity and quality by altered plant inputs in soil, such as litter and rhizodeposition (DeVries et al., 2019; Karlowsky et al., 2018; Kuzyakov, 2002; Sánchez‐Cañizares et al., 2017; Sasse et al., 2018; Williams & DeVries, 2020). Drought‐induced changes of rhizodeposition strongly depend on species identity and drought intensity (Preece & Peñuelas, 2016) and can alter nutrient availability through shifts in fungi/bacteria ratios, causing shifts in plant composition (Peguero et al., 2019; Preece & Peñuelas, 2016). Drought also reduces litter quality and thereby leads to lower mineralization rates. The resulting deceleration of nutrient cycling and the enhancement of fungal dominance in the microbial community in turn can alter plant community composition and favour more drought adapted species (Pugnaire et al., 2019). Finally, drought legacies not only affect PSFs between species but also within species, by favoring genotypes within plant species that develop less negative feedback and thereby decreasing intraspecific diversity (Crawford & Hawkes, 2020).

Drought legacies have been shown to lead to reduced leaf area index in grasslands and forest (Jiao et al., 2021; Kannenberg, Novick, et al., 2019) and to affect the *canopy structure* (Beloiu et al., 2022), driven by changes in species abundance and composition, for example in forests subjected to wide‐spread mortality (Saatchi et al., 2013; Senf et al., 2021). Changes in canopy structure can alter abiotic ecosystem properties such as light availability and *microclimate*, with consequences for the composition and biodiversity of the understory as well as nutrient and C cycling (Kane et al., 2011; Royer et al., 2011; Anderegg et al., 2012, 2013). Drought can have a positive or negative legacy effect on soil moisture in grasslands, lasting up to a half year post‐drought (Robinson et al., 2016; Reinthaler et al., 2021; Hoover et al., 2021). Positive soil moisture legacies can be driven by a post‐drought decrease of species with low drought resistance, which can reduce community‐level water demand (Hoover et al., 2021). Drought can also cause legacy effects on *soil properties*, by altering the chemical and physical soil structure. Drought has been shown to increase the soil water repellency (Goebel et al., 2011; Sánchez‐García et al., 2019), decrease soil moisture retention and soil moisture storage capacity (Robinson et al., 2016). It can also change aggregate stability (Goebel et al., 2005) with cascading effects on ecosystem functioning. For example, an increase in soil water repellency caused by drought can reduce the mineralization of soil organic matter by microbes with potential consequences for plant productivity and plant community structure (Goebel et al., 2011).

## 4 DROUGHT LEGACY DURATIONS

**FIGURE 3:**
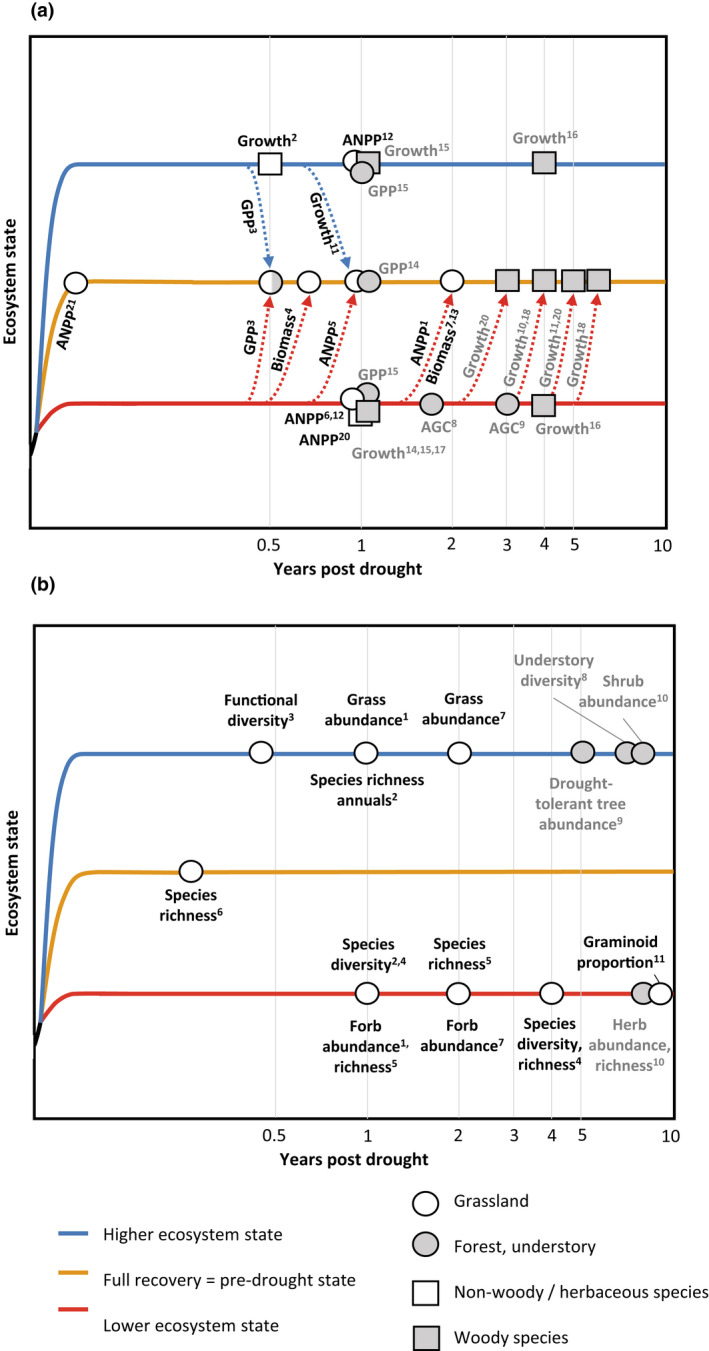
Drought legacies durations of (a) carbon‐cycle parameters and (b) community properties (species abundance, composition, and richness) for different plant functional types and ecosystems, respectively. Abbreviations for 3a: Asat = light saturated photosynthetic exchange rate, growth = in forest/woody species this refers to radial growth/tree ring width, ANPP = aboveground net primary production, GPP = gross primary productivity, AGC = aboveground carbon stocks. References for (a) are indicated as follows: 1. Xu et al. (2021), 2. Hahn et al. (2021), 3. Xie et al. (2020), 4. Mackie et al. (2019), 5. Stampfli et al. (2018), Hoover et al. (2014), 6. Sala et al. (2012), 7. DeBoeck et al. (2018b), 8. Wigneron et al. (2020), 9. Yang et al. (2018), 10. Anderegg, Schwalm, et al. (2015), 11. Wu et al. (2018), 12. Griffin‐Nolan et al. (2018), 13. Xu et al. (2017), 14. Kannenberg, Novick, et al. (2019), 15. Kannenberg et al. (2020), 16. Peltier et al. (2016), 17. Gazol et al. (2020), 18. Itter et al. (2019), 19. Szejner et al. (2020), 20. Hoover et al. (2021), 21. Gao et al. (2021). For related physiological parameters see also Ruehr et al. (2019). References for (b) are indicated as: 1. Hoover et al. (2014), 2. Stampfli et al. (2018), 3. Griffin‐Nolan et al. (2019), 4. Xu et al. (2017), 5. DeBoeck et al. (2018b), 6. Gao et al. (2021), 7. Xu et al. (2021), 8. Kane et al. (2011), 9. Suarez and Kitzberger (2008), 10. Anderegg et al. (2012), 11. Stampfli and Zeiter (2004).

To date few studies have explicitly looked into drought legacy duration, which has been best documented for C cycle processes. Here, we synthesize drought legacy duration post‐drought for a range of C cycle parameters and for community properties, which both strongly depend on the plant functional types and the specific response parameter studied (Figure 3).

In grasslands, most C‐cycle related legacies return to pre‐disturbance level roughly within the first year after the drought and can last several years for forests (Figure 3a). This is in line with the suggestion by Wu et al. (2018), and Zhang et al. (2022) that drought legacies tend to be longer for forest and woody species compared to grasslands and non‐woody/herbaceous species.

Overall, flux parameters return to pre‐disturbance levels within the first year (Figure 3a, see also Schwalm et al., 2017; Zhao et al., 2020), while biomass‐ and growth‐related legacies tend to persist long afterwards (Figure 3a). This supports the emerging notion of a post‐drought decoupling of temporal dimensions of response parameters in forests such as C uptake, tree rings, and NDVI (Kannenberg, Novick, et al., 2019; Gessler et al., 2020; Gazol et al., 2020; Kannenberg et al., 2020), showing that the legacy duration of different C cycle response parameters is highly variable.

Furthermore, we observed that legacies in community properties, such as species abundance, composition, and richness tend to last longer in woody species and understory compared to grasslands (see Figure 3b). Moreover, the drought legacy effects on community properties tend to last longer than those related to C cycle parameters (Figure 3). For example, while biomass recovered after drought in a grassland experiment (Figure 3a), species composition still remained affected after one (Hoover et al., 2014) and 2 years (DeBoeck et al., 2018; Xu et al., 2021; Figure 3b). Following severe drought events, community properties often do not return to pre‐disturbance levels (Figure 3; Hillebrand & Kunze, 2020).

Overall, the temporal aspect of drought legacies and their dependencies are still poorly understood across response parameters and plant functional types. This is especially relevant for long‐term legacies that are related to community properties (Hillebrand & Kunze, 2020; see Figure 3b). By conducting continuous measurements long after the drought has subsided and thereby revealing when deviations of response parameters return to the baseline, studies could provide insight into the duration and cumulative magnitude of drought legacies. Based on the scarce available evidence, we suggest that to fully quantify drought legacies, observations of up to 5 and 15 years may be required for grasslands and forests, respectively.

## 5 EFFECTS OF DROUGHT LEGACIES ON RESPONSES TO SUBSEQUENT DROUGHT EVENTS

While legacies after a drought event have been increasingly studied in recent years, we still lack a profound understanding of how these drought legacies alter the resilience (i.e. resistance and recovery [*sensu* Ingrisch & Bahn, 2018]) of ecosystems to subsequent droughts (or other extreme events, see e.g. Zscheischler et al. 2018). Drought legacy effects on ecosystem responses of a subsequent drought can relate to all ecosystem properties and processes (intrinsic factors, IFs) outlined above. In the following, we develop hypotheses about the main determinants of the resilience trajectories of an IF to subsequent drought events.

**FIGURE 4:**
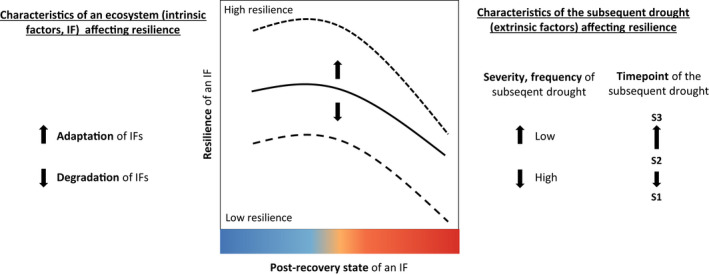
Hypothesized changes in ecosystem resilience of an ecosystem property or process (intrinsic factor, IF) to a subsequent drought in relation to (i) its post‐recovery state following the antecedent drought event, (ii) the adaptation versus degradation of other ecosystem properties and processes (IFs) as well as iii) characteristics of the subsequent drought. The color code of post‐recovery state refers to Figure 1, blue and red indicating an increase or decrease in ecosystem state, respectively. Next to the post‐recovery state, adaptation and degradation of IFs (for a summary of IFs, see Figure 2, for examples on adaptations and degradations of IF see Figure 5) can alter resilience to subsequent drought. Extrinsic factors, including timing (S1–S3, see Figure 1), the severity, and the frequency of the subsequent drought(s) can affect resilience (defined here as the combined resistance to and recovery from a drought event).

First, we hypothesize that the resilience of an IF to a subsequent drought depends on its *post‐recovery state* following the antecedent drought event. Relations can be manifold and depend on the particular IF, hence for simplicity we only present one option here, showing the highest resilience when the IF reveals no legacy from the previous drought (Figure 4).

**FIGURE 5:**
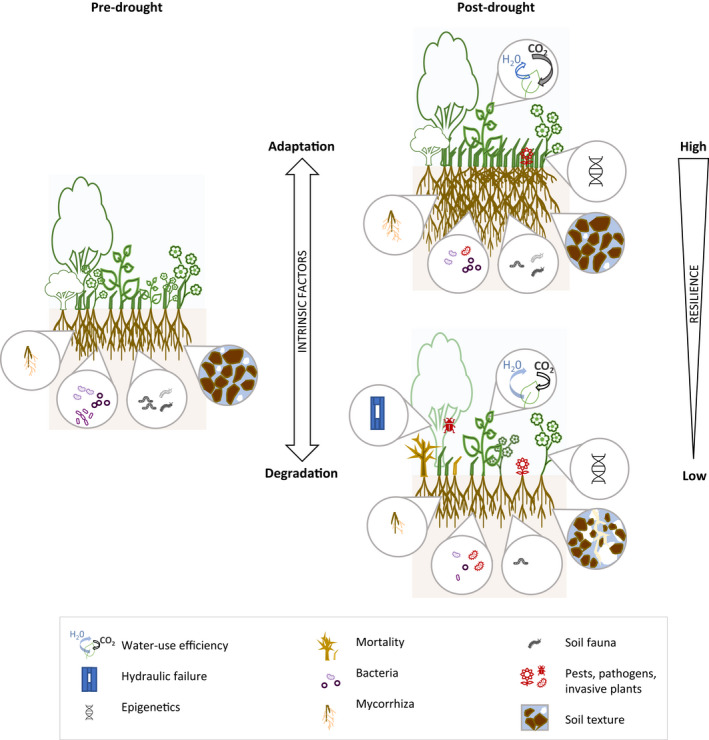
Post‐drought adaptation/degradation of selected processes and properties on species, community, ecosystem scale (intrinsic factors) associated with higher/lower resilience (i.e. capacity to resist and recover) toward a subsequent drought event. An adaptation, e.g. through increases in fine root mass, mycorrhizae or water use efficiency (CO_2_ uptake relative to H_2_O loss), will lead to higher resilience, while a degradation, e.g. of plant cover, species diversity or soil texture, will typically lead to a lower resilience. For further explanations see the text.

Second, we suggest that the resilience of an IF to a subsequent drought depends on the *adaptation* and *degradation* of all further IFs of the ecosystem (Figures 2 and 4). We hypothesize that post‐drought legacy adaptation/degradation of all further IFs of an ecosystem can shift the response of an IF to a subsequent drought toward higher/lower resilience, respectively (Figures 4 and 5). Importantly, different IFs can be affected by adaptations and degradations to different degrees (Figure 5).

On the species scale, *adaptations* imply that species become more adjusted to drought, which can moderate the impact of a subsequent drought event. For example, a reduced xylem conduit size in trees can reduce the risk of hydraulic failure and thereby increase resistance to a subsequent drought (Gessler et al., 2020). Proline, a water retaining compound that can regulate osmotic adjustments, was found to be adaptively enriched in grassland species under recurrent drought conditions (Li et al., 2022). The observed higher water retention and concurrent higher stomatal conductance can maintain plant functioning during subsequent droughts (Li et al., 2022). Furthermore, an adaptation toward higher photosynthetic rate was observed under recurrent drought and during its recovery (Alves et al., 2020). Moreover, a higher root biomass as a legacy from a previous drought can increase resistance and recovery to a new drought (Legay et al., 2018). It is known that molecular mechanisms such as accumulation of proteins and transcription factors, as well as epigenetic changes can change plant responses to subsequent stress (Bruce et al., 2007; Jacques et al., 2021). For example, drought was suggested to result in epigenetic changes leading to structural changes (Fleta‐Soriano & Munné‐Bosch, 2016) or enhanced transcription of stress‐response genes (Ding et al., 2012), thereby increasing plant resistance to a subsequent drought. These mechanisms of ‘drought memory’ (Walter et al., 2013) were hypothesized to be an underlying cause for increased resistance of plant biomass during subsequent drought (Walter et al., 2011; Backhaus et al., 2014).

Long‐term adaptations on the community scale have been documented for all plant functional types. Such adaptations frequently involve increased dominance of drought adapted species (Hoover et al., 2014; DeBoeck et al., 2018; Xu et al., 2021; Wilcox et al., 2021) and lead to corresponding shifts in the community‐level plant traits (Trugman et al., 2020; Evans et al., 2022). They have also been shown to involve increases in functional diversity (Griffin‐Nolan et al., 2019). Such community‐level adaptations can moderate the impact of a subsequent drought (Coleman & Wernberg, 2020; see Figure 5). For example, an increase of trees with drought‐tolerant hydraulic traits can buffer forest productivity during subsequent droughts (Trugman et al., 2020). Moreover, an adaptation of soil biota and processes can dampen the negative effect of a subsequent drought on native plant species while reducing the success of invasive species (Meisner et al., 2013). It has recently also been shown that multiple recurrent droughts can alter soil microbial community composition and enhance soil multifunctionality during subsequent drought events (Canarini et al., 2021).

On the ecosystem scale, increased N availability upon rewetting can favor resistance to and recovery from subsequent drought (Legay et al., 2018). Recurrent drought events have been shown to enhance such rewetting‐induced N release both in the lab (Miller et al., 2005; Lu et al., 2019) and in the field (DeVries et al., 2012). However, several studies also suggest that under recurrent droughts this rewetting effect can be dampened (Borken & Matzner, 2009; Yu et al., 2014; Kaisermann et al., 2017; Sánchez‐García et al., 2019), which might lead to an overall reduction of N availability in the ecosystem, as rewetting can fail to balance the decreased N mineralization rates during drought events (Borken & Matzner, 2009) or lead to enhanced N leaching (Sardans et al., 2020; Krüger et al., 2021).

In addition to drought‐induced adaptations, *degradations* of intrinsic factors can have an important influence on ecosystem responses to subsequent droughts. In fact, it has been suggested that an increasing amount of land area globally may be degraded by aridity in the long‐term due to shifts in precipitation regimes (Berdugo et al., 2020), activating a range of dryland mechanisms (Grünzweig et al., 2022). Increased aridity can hamper the recovery after a drought event and lead to more extreme responses to recurrent drought events. Degradation can involve both plant‐ and soil‐related parameters such as plant cover and soil aggregate stability (Berdugo et al., 2020). Furthermore, legacies in fungi/bacteria ratio can decrease the ability of soil microbial communities to maintain the same functions under recurrent drought (Preece et al., 2019). Degradation can also imply reduced biodiversity (Jung et al., 2014; Hoover et al., 2014; Xu et al., 2017; Stampfli et al., 2018), which is an important stabilizing factor for ecosystem productivity and both increases the resistance to (Isbell et al., 2015) and recovery from drought (van Ruijven & Berendse, 2010; Kreyling et al., 2017; Craven et al., 2018). Moreover, negative effects on seedbanks can affect plant communities and could reveal themselves after a long time, as they are often not reflected in the aboveground vegetation (Basto et al., 2018).

Finally, we hypothesize that the resilience trajectories of an IF to a subsequent drought event are strongly influenced by extrinsic factors, including drought timing, frequency, and severity (Figure 4). Next to seasonality effects, timing matters for the degree of the recovery from the previous drought (Figure 4). Overall, we expect that resilience is lower when the species, community, or ecosystem property or process has not yet recovered from the previous drought (Figure 1, S1 and S2) and higher when it is fully recovered (Figure 1, S3; Mitchell et al., 2016; Schwalm et al., 2017; Peltier & Ogle, 2019; Szejner et al., 2020; Hoover et al., 2021). Furthermore, resilience to a subsequent drought is probably decreased by drought frequency, that is the number of consecutive drought events. Several studies in fact support the notion that a higher drought frequency decreases both resistance (Bose et al., 2020; Xu et al., 2021) and recovery (Gao et al., 2018; Peltier & Ogle, 2019; Szejner et al., 2020; Jiao et al., 2021; Serra‐Maluquer et al., 2021). However, the opposite, that is a higher drought frequency leading to a higher resilience, has also been shown (Yao et al., 2022; see also the above section on adaptations shaping the resilience to a subsequent drought event). Also, increasing drought severity is expected to decrease resistance to and recovery from a subsequent drought (Figure 4). This hypothesis is based on studies of single drought events, where longer duration hampered resistance (Buttlar et al., 2018; Reynaert et al., 2020), and higher intensity reduced resistance (Xu et al., 2019) and recovery (Schwalm et al., 2017). Given the broad lack of evidence on the interactive effects of intrinsic and extrinsic factors, experimental and observational studies are urgently needed to improve our understanding of ecosystem responses to recurrent drought events.

## 6 CONCLUSION AND OUTLOOK

In times of increasing severity and frequency of drought events in many parts of the world, it is essential to not only assess the concurrent effects of droughts, but to understand the lasting consequences such extreme events may have on ecosystems. In our review, we have provided a broad overview of drought legacies and the underpinning mechanisms from species to community and ecosystem scale. To date, quantitative analyses of drought legacy responses have mainly focused on aboveground growth‐related parameters and some community attributes, suggesting that the legacy duration can differ vastly for different parameters and different plant functional types. For a more in‐depth understanding of drought legacies on ecosystems, it will be important for future studies to extend the observational timescale and explicitly consider a range of interrelated biotic and abiotic factors, including above‐belowground interactions. To advance the field, it will be essential to illuminate the particular role of adaptation and degradation of properties and processes across scales in determining ecosystem resilience to subsequent drought events. Furthermore, future studies should consider potential interactions of drought legacies with other global change factors such as warming, elevated CO_2_, N deposition and land‐use changes, as well as interactions with other climate extremes, such as heatwaves and heavy precipitation events. Accounting for these potential interactions and the implications of drought legacies for subsequent drought events is essential for understanding and projecting the long‐term consequences of a changing climate for ecosystems.

## CONFLICT OF INTEREST

The authors declare no conflict of interest.

## Supporting information


Appendix S1.

## Data Availability

Data sharing is not applicable to this article as no new data were created or analyzed in this study.
