# Supplementary material for: Drought legacies and ecosystem responses to subsequent drought
Source: Glob Chang Biol. 2022 Jun 23;28(17):5086–103. doi: 10.1111/gcb.16270 (PMC9542112; doi:10.1111/gcb.16270)
Supplement: Supplementary file 1 — Appendix S1. [file GCB-28-5086-s001.pdf]

Supporting Information

Fig. S1 gives a summary of peer-reviewed publications that studied drought legacies on species-, community- and ecosystem scale. This overview is based on a literature search in the Web of Science performed in April 2022, using all databases and searching the following keywords: ‘drought*’ in title AND ‘legac*’, ‘lag*’, ‘memory’, ‘resilience’, and ‘recovery’ in the title and the abstract. Given the focus of this review on natural and semi-natural systems we excluded the term ‘cropland*’ in the title. We also excluded papers using the terms ‘warming’ and ‘elevated CO_2’ in the title. Within the 3504 papers obtained we searched for the terms listed in the caption of Fig. S1, allocated to the different scales shown in Fig. 2 and to different ecosystem types/plant functional types (grassland/grass*/forb*/herb*; shrubland/shrub*; forest/tree*). Of the 3292 entries displayed across the topics, 30.3 % were allocated to the category grassland (including grass*, forb* and herb*), 7.3 % of the papers to shrubland (including shrub*), and 62. 4 % of the papers were allocated to the category forest (including tree*).

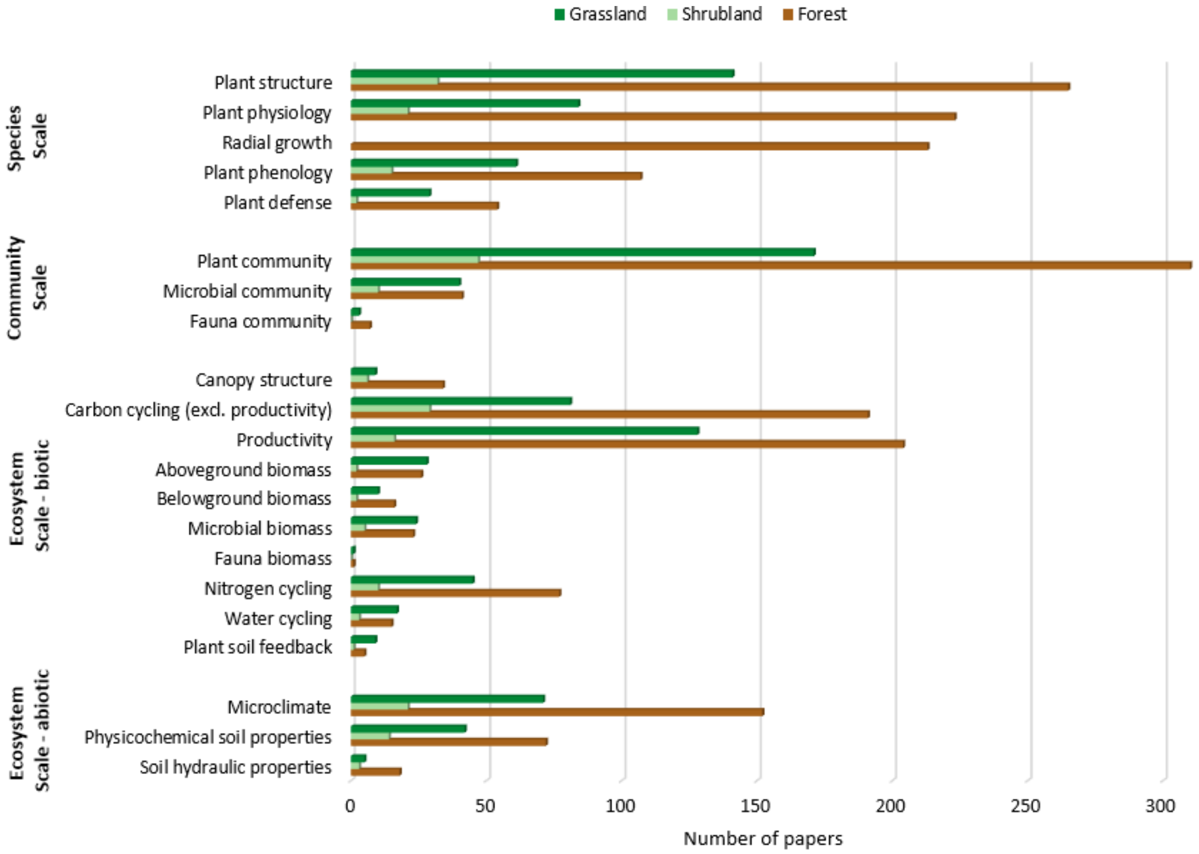

Fig. S1: Number of papers reporting on drought legacies on species, community, and ecosystem scale presented for grasslands (dark green), shrublands (light green), and forests (brown) and the respective species / communities. The following terms were searched within all fields (terms were combined with OR unless mentioned otherwise):

Species Scale: Plant structure: leaf N, leaf nitrogen, leaf trait*, root N, root nitrogen, root trait*, stolon*, structure, tiller*; **Plant physiology**: epigenetic*, growth rate, physiology, signal* metabolite*, transcription, water uptake; **Radial growth for forest/tree*:** basal increment growth, radial growth, tree ring*; **Plant phenology**: flowering date, flowering length, growing season, growth onset, phenology; **Plant defense**: herbivory, pathogen*, pest*, plant defence, plant defense

Community Scale: Plant Community: plant* AND abundance, community, composition, diversity, hydraulic trait*, invasive, mortality, richness, seedbank; **Microbial community**: archaea, bacteria*, fung*, microb* AND abundance, community, composition, diversity, invasive, mortality, richness; **Fauna**: fauna* AND abundance, composition, diversity, invasive, mortality, richness

Ecosystem scale – Biotic: **Canopy structure:** canopy structure, LAI; **Carbon cycling (excl. productivity)**: C cycling, carbon cycling, C dynamics, carbon dynamics; **Productivity:** GPP, gross primary productivity, productivity; **Aboveground biomass:** aboveground biomass, ANPP; **Belowground biomass:** belowground biomass, root* mass, BNPP; **Microbial biomass**: bacterial biomass, fung* biomass, microbial biomass; **Fauna biomass**: fauna biomass; **Nitrogen cycling**: N cycling, nitrogen cycling, N dynamics, nitrogen dynamics; **Water cycling:** water cycling; **Plant soil feedback**: plant soil feedback, PSF

Ecosystem Scale – Abiotic: **Microclimate**: light availability, microclimate, radiation, soil moisture; **Physicochemical soil properties**: aggregate stability, soil propert*, soil structure; **Soil hydraulic properties**: hydrophob*, soil hydraulic propert*, water repellency, water retention
